# Novel Quorum Quenching YtnP Lactonase From *Bacillus paralicheniformis* Reduces *Pseudomonas aeruginosa* Virulence and Increases Antibiotic Efficacy *in vivo*

**DOI:** 10.3389/fmicb.2022.906312

**Published:** 2022-06-02

**Authors:** Lidija Djokic, Nada Stankovic, Ivana Galic, Ivana Moric, Natasa Radakovic, Sandra Šegan, Aleksandar Pavic, Lidija Senerovic

**Affiliations:** ^1^Institute of Molecular Genetics and Genetic Engineering, University of Belgrade, Belgrade, Serbia; ^2^Institute of Chemistry, Technology and Metallurgy, National Institute of the Republic of Serbia, University of Belgrade, Belgrade, Serbia

**Keywords:** anti-virulence, anti-biofilm, *N*-acyl-homoserine lactones, opportunistic pathogens, zebrafish

## Abstract

Bacterial infections have become increasingly difficult to treat due to the occurrence of antibiotic-resistant strains. A promising strategy to increase the efficacy of therapy is to combine antibacterials with agents that decrease pathogen virulence *via* the modulation of the quorum sensing (QS). Lactonases inhibit acylated homoserine lactone (AHL)-mediated QS in Gram-negative bacteria, including the leading nosocomial pathogen *Pseudomonas aeruginosa*. Here we describe the characteristics of heterologously expressed YtnP lactonase from *Bacillus paralicheniformis* ZP1 (YtnP-ZP1) isolated from agricultural soil using the culture enrichment method. Purified YtnP-ZP1 hydrolyzed different AHLs with preference to substrates with long acyl residues as evaluated in assays with biosensors and HPLC. The enzyme showed good thermostability and activity in a wide temperature range. YtnP-ZP1 in 50 μg mL^–1^ concentration reduced the amount of *P. aeruginosa*-produced long-chain AHLs by 85%, while it hydrolyzed 50% of short-chain AHLs. Incubation of *P. aeruginosa* PAO1 with YtnP-ZP1 reduced its swarming motility and elastolytic activity without bactericidal effect. YtnP-ZP1 caused the inhibition of biofilm formation and disintegration of mature biofilms in *P. aeruginosa* PAO1 and multiresistant clinical strain BR5H that was visualized by crystal violet staining. The treatment with YtnP-ZP1 in concentrations higher than 25 μg mL^–1^ improved the survival of *P. aeruginosa* PAO1-infected zebrafish (*Danio rerio*), rescuing 80% of embryos, while in combination with tobramycin or gentamicin survival rate increased to 100%. The treatment of *P. aeruginosa* PAO1 biofilms on infected zebrafish tail wounds with 50 μg mL^–1^ YtnP-ZP1 and 2 × MIC tobramycin led to infection clearing in 2 days. The extensive toxicity studies proved YtnP-ZP1 was non-toxic to human cells and zebrafish. In conclusion, novel YtnP-ZP1 lactonase with its effective anti-virulence activity could be used to increase the efficacy of clinically approved antibiotics in clearing both systemic and biofilm-associated *P. aeruginosa* infections.

## Introduction

The rapid emergence and spread of multidrug-resistant (MDR) pathogens present a global healthcare challenge. Of special concern are human and animal bacterial pathogens that have advanced into MDR forms following antibiotics use (Davies and Davies, 2010). Moreover, new opportunistic pathogenic strains with low susceptibility to antibiotics are emerging outside the clinical or farm settings, demonstrating that other environments are also significant sources of antibiotic-resistant bacteria (Munita and Arias, 2016; Algammal et al., 2020a,b). The development of resistance at the cellular level is a result of mutations in genes that are often associated with the mechanism of action of the antibacterial agent or through horizontal gene transfer of resistance determinants from another microorganisms. However, additional mechanisms also help bacteria to survive exposure to antibiotics such as tolerance, i.e., the ability of bacteria to survive in the presence of antibiotics without developing resistance (Levin-Reisman et al., 2017). One of the common causes of resistance and/or tolerance to antibiotics are biofilms (Hall and Mah, 2017), complex communities of bacteria embedded in a self-produced matrix of polysaccharides, proteins, and extracellular DNA (Hall-Stoodley et al., 2004). In biofilms, bacterial cells can be up to 1,000 times less susceptible to the antibacterial agents compared with their free-living forms (Balcázar et al., 2015). Some of the mechanisms responsible for biofilm resistance to antibiotics include the production of molecules sequestering antibiotics, matrix β-lactamases, and antibiotic efflux pumps, while biofilm tolerance is often a result of reduced growth rate, presence of persister cells, and the mechanisms dealing with antibiotic-induced oxidative stress (Olsen, 2015; Hall and Mah, 2017).

Biofilm formation and maturation are regulated by quorum sensing (QS), a cell density-dependent communication system that relies on the synthesis, diffusion, and detection of small signaling molecules, also known as autoinducers (AIs) (Waters and Bassler, 2005). Besides biofilm, an arsenal of virulence factors, diverse in structure, function, and localization, which are important for the establishment of bacterial pathogenicity, are also under QS control.

As antibiotic resistance has become a major concern in human healthcare, quorum sensing interference (QSi) has been suggested to be a promising alternative strategy to fight bacterial infections (Chen et al., 2013). Rather than targeting bacterial growth or viability as in antibiotic therapy, QSi targets bacterial virulence machinery required for host damage and disease and suppresses the production of virulence factors, including biofilm formation. Non-bactericidal QSi agents are aimed to disarm bacteria and reduce their pathogenicity allowing the host immune system and its normal microbial flora to prevent pathogen colonization and eradicate the established infection. They are intended to be used in combination therapy with clinical antibiotics, rendering pathogenic bacteria more sensitive to applied antibiotics, and thus restoring the efficiency of currently available therapeutics (Rezzoagli et al., 2020).

To accomplish QSi two main approaches are used which include (i) preventing bacteria to produce or perceive AIs, and (ii) degradation of AIs (Geske et al., 2008; Galloway et al., 2011). The first strategy is mainly based on the identification of (small) molecules that inhibit QS by different mechanisms. In order to act, QS inhibitors need to bind to an intracellular target protein. Since anti-virulence agents, in general, do not kill bacteria, it is believed that this alternative strategy induces a milder selection pressure than antibiotics. Still, there is some evidence which shows that resistance toward small molecules inhibiting QS can be developed, but that would be spread slowly. The second QSi approach, which involves the use of quorum quenching (QQ) enzymes that cut AIs, is most likely the least resistance-prone anti-virulence strategy as QQ enzymes act remotely and independently and do not need to enter the bacterial cell. Moreover, enzymes are usually non-toxic and may be immobilized on various matrices without being released, and as such they are highly attractive for the development of novel alternative antibacterials (Bzdrenga et al., 2017).

World Health Organization (WHO) has recently flagged several Gram-negative antibiotic-resistant strains as research priority targets for the development of new therapeutics, including MDR *Pseudomonas aeruginosa* (World Health Organization, 2017). *P. aeruginosa* can be a part of the transient microbiota of the skin and the mucous membranes of healthy individuals, but it can lead to infections and serious illness following the discontinuation of skin and mucous membranes (wounds caused by burns, surgical wounds, catheterization, or patient intubation), immunodeficiency (cystic fibrosis, cancer, AIDS), as well as changes in saprophytic microbiota due to the use of antibiotics (Lister et al., 2009). Although the ability to form biofilms is the major virulence characteristic of *P. aeruginosa*, other virulence factors such as phenazines, elastase, phospholipases, rhamnolipids, exotoxins, and endotoxins, whose secretion is mediated by QS, are important for the pathogenicity of this bacterium.

In wide range of Gram-negative bacteria, including *P. aeruginosa*, *N*-acyl homoserine lactone (AHL) is one of signaling molecules used in interbacterial communication, and thus represent a suitable target for the development of anti-virulence agents. An AHL molecule scaffold consists of homoserine lactone (HSL) ring and an acyl chain whose length and nature may vary (Czajkowski and Jafra, 2009). Some enzymes are naturally capable of modifying or degrading these signaling molecules, such as AHL acylase, AHL lactonase, oxidoreductase, and paraoxonase (Chen et al., 2013). Since stability is the major constraint that usually impairs enzyme utilization, intensive efforts have been dedicated for the isolation of robust enzymes from extreme and hostile environments.

In the quest for safe, least resistance-prone, and sturdy antibacterial agent, in this study, we aimed to isolate a stable QQ enzyme with anti-virulence activity that could be used in combination with clinical antibiotics to achieve efficient treatment of *P. aeruginosa* infections.

## Materials and Methods

### Microbial Strains

*Pseudomonas aeruginosa* PAO1 DSMZ 22644 was used to evaluate anti-virulence activity, *P. aeruginosa* PA14 was used to measure pyocyanin production, *P. aeruginosa* BR5H was used to evaluate anti-biofilm activity (Milivojevic et al., 2018), *P. aeruginosa* PAOJP2/pKD-*rhlA* (Δ*rhlA* P*rhlA:lux*) was used to measure short-chain AHLs (Duan and Surette, 2007); *P. aeruginosa* PA14-R3 (ΔlasI P*rsal*:*lux*) was used to measure long-chain AHLs (Massai et al., 2011), and *P. aeruginosa* PAO1 containing pBBR2-GFP was used to visualize infections *in vivo* (Passos da Silva et al., 2014). *Chromobacterium violaceum* CV026 (McClean et al., 1997), recently reclassified as *C. subtsugae*, was used to detect medium-chain AHLs (Harrison and Soby, 2020).

### Isolation and Characterization of Bacteria From Environmental Samples

#### Isolation of Quorum Quenching Bacteria

A sample of soil was obtained from an agricultural plot heavily treated with aromatic chlorinated herbicides in Zemun Polje, Serbia (44°86′90.8′′N, 20°33′30.2′′E). A total of 0.5 g of soil was resuspended in 4.5 mL of MM medium [0.7 g K_2_HPO_4_, 2 g KH_2_PO_4_, 0.1 g MgSO_4_ × 7H_2_O, 1 g (NH_4_)_2_SO_4_, 0.5 g peptone, 100 g NaCl per 1 L, pH 7.0], thoroughly mixed, and incubated for 30 min at 30°C on a rotary shaker at 150 rpm. The soil suspension (1% v/v) was used to inoculate MM medium supplemented with 2 mM *N*-octanoyl-L-homoserine lactone (C8-HSL; Sigma-Aldrich, United States) as a sole carbon source and incubated at 30°C for 72 h on a rotary shaker at 180 rpm. The culture was subinoculated (1% v/v) in the same medium containing C8-HSL at 3-day intervals. After the third enrichment cycle, 200 μL of cell suspension was spread on lysogeny broth (LB), Brain-Heart Infusion (HiMedia, Thane West, Maharashtra, India), and Plate Count Agar (HiMedia, Thane West, Maharashtra, India) solid media to obtain pure colonies. After incubation for 24 h at 30°C, colonies with different morphology were selected.

#### Selection of Biofilm-Forming Environmental Bacteria

Biofilm-forming environmental bacteria were selected from the in-house bacterial strains collection that consists of chlorinated herbicide-tolerant bacteria based on crystal violet staining in static biofilm formation assay described below.

### 16S rDNA and Whole Genome Sequencing

#### Identification of Environmental Bacteria Isolates

The genomic DNAs (gDNA) isolated using GeneJET Genomic DNA Purification Kit (Thermo Fisher Scientific, Waltham, MA, United States) were used for amplification of the genes coding for 16S rRNAs using bacteria-specific primers, 27F and 1492R (Invitrogen, Waltham, MA, United States) (Lane, 1991), and FastGene Taq DNA Polymerase (Nippon Genetics, Duren, Germany). PCR products were purified with QIAquick PCR purification kit (Qiagen, Hilden, Germany), sequenced using BigDye™ Terminator v3.1 Cycle Sequencing Kit (Applied Biosystems, Waltham, MA, United States), and run on Applied Biosystems^®^ 3130 Genetic Analyzer (United States). Sequences were analyzed and assembled with SeqMan tool from DNASTAR Lasergene Software (DNASTAR, Madison, WI, United States). BLASTn program (Altschul et al., 1990) was used to perform a similarity search in the GenBank database provided by the NCBI.

#### Whole-Genome Sequencing and Analysis

Total gDNA of the isolate ZP1 was sequenced on Illumina HiSeq 2500 platform in MicrobesNG (Birmingham Research Park, Birmingham, United Kingdom). Genome assembly was performed using De Bruijn Graph methods and contigs shorter than 200 base pairs (bp) were eliminated. Burrows-Wheeler Aligner (BWA) was applied to map raw reads to assembled scaffolds. Rapid Annotations using Subsystems Technology (RAST) server^1^ were used for the prediction of open reading frames and gene annotation.

### Lactonase Expression and Purification

The coding sequence of *ytnP* lactonase was amplified using Phusion Hi-Fidelity Polymerase (Thermo Fisher Scientific, Waltham, MA, United States) and primers (Invitrogen, Waltham, MA, United States) listed in Supplementary Table 1 with gDNA of ZP1 isolate DNA as the template, according to manufacturer’s instructions. Amplified *ytnP* gene was cloned without purification into the pETite C-His Kan plasmid, and chemically competent *Escherichia coli* HI-Control 10G (Lucigen, Middleton, WI, United States) were transformed. The presence of the insert was confirmed by sequencing with T7 promoter and pETite reverse primers (Supplementary Table 1), using the procedure described in 2.4.1, and pETite vector carrying *ytnP* was subsequently transformed in *E. coli* BL21 (DE3) expression cells (HiMedia, Thane West, Maharashtra, India).

LB medium supplemented with 30 μg mL^–1^ kanamycin and 1% glucose was inoculated with confirmed construct and grown for 16 h at 37°C and 180 rpm. The overnight culture was used to inoculate 200 mL Terrific Broth medium (HiMedia, Thane West, Maharashtra, India) (2%, v/v) supplemented with 30 μg mL^–1^ kanamycin and incubated (37°C, 180 rpm) until the optical density at 600 nm reached values of 0.4–0.6. The expression of lactonase was induced with 1 mM IPTG and the culture was incubated at 17°C for 72 h with shaking at 180 rpm. After incubation, cells were pelleted (4500 × *g*, 20 min, 4°C), then resuspended in 10 mL wash buffer (50 mM NaH_2_PO4, 300 mM NaCl, with 10 mM imidazole, pH 7.4) with lysozyme, and finally disrupted by sonication on ice (4 pulses of 20 s at 60 kHz, with 20 s of cooling between each pulse). The cell lysate was filtered, loaded on Ni-NTA agarose (Qiagen, Hilden, Germany) column equilibrated with wash buffer, and His-tagged protein was washed using imidazole gradients of washing and elution buffers (50 mM NaH_2_PO4, 300 mM NaCl, imidazole 20, 50, 100, 150, and 250 mM imidazole, pH 7.4). Protein was eluted in elution buffer with 250 mM imidazole and the buffer was exchanged with 20 mM sodium phosphate buffer (0.011 M Na_2_HPO_4_ × 7H_2_O, 0.008 M NaH_2_PO_4_ × H_2_O, pH 7.0) and concentrated using Millipore centrifugal filter unit Ultracel-10K (Milipore, Burlington, MA, United States). Protein content was measured by Bradford method (Bradford, 1976) and protein purity was verified by SDS-PAGE electrophoresis (10%) after staining with Coomassie Brilliant Blue R-250. Blue Star protein standard mixture from Nippon Genetics (Duren, Germany) was used as the reference.

### Assessment of Quorum Quenching Activity of Bacteria

To examine the QQ activity of ZP1 isolate an overnight bacterial culture in LB was washed three times with PBS pH 7.2–7.4 and then resuspended in PBS containing 10 μM *N*-(3-oxodecanoyl)-L-homoserine lactone (3oxo-C12-HSL; Sigma-Aldrich, St. Louis, MO, United States), 10 μM *N*-(hexanoyl)-L-homoserine lactone (C6-HSL; Sigma-Aldrich, St. Louis, MO, United States), or *N*-butanoyl-L-homoserine lactone (C4-HSL; Sigma-Aldrich, St. Louis, MO, United States). Cultures were incubated for 20 h at 30°C on a rotary shaker at 180 rpm. After centrifugation, supernatants containing AHLs were filtered and the filtrates were used for the evaluation of AHL levels either in *C. violaceum* disk assay or in assays with *P. aeruginosa* biosensors. Control samples containing 10 μM AHLs in PBS were either immediately placed at 4°C and used to quantify the starting amount of AHLs or incubated under the same conditions as samples with bacteria and used as control of spontaneous degradation.

#### CV026 Disk Assay

Aliquots of 10 μL of filtrate containing C6-HSL were applied to cellulose disks placed on LB soft agar inoculated with *C. violaceum* CV026 and overlaid on LB agar. After incubation for 20 h at 30°C, a decrease in C6-HSL concentration was detected as reduced purple zone of violacein produced around the disks.

#### Assay With *Pseudomonas aeruginosa* Biosensors

Overnight cultures of biosensors *P. aeruginosa* PA14-R3 (Δ*lasI* P*rsal:lux*) or PAOJP2/pKD-*rhlA* (Δ*rhlA* P*rhlA:lux*) were diluted in LB to *OD*_600_ = 0.045 and incubated with lactonase-treated 3oxo-C12-HSL or C4-HSL, respectively (Duan and Surette, 2007; Massai et al., 2011). After 4 h of incubation at 37°C, cell density (*OD*_600_) and luminescence (light counts per second, LCPS) were simultaneously measured using Tecan Infinite200 (Tecan, Mannedorf, Switzerland). Luminescence values were normalized per cell density. The assay was carried out in quadruplicate and repeated at least three times.

### Assessment of Lactonase Activity

The activity of purified YtnP-ZP1 was assessed by incubating the enzyme with 10 μM 3oxo-C12-HSL or 10 μM C4-HSL in sodium phosphate buffer for 20 h at 30°C, and the next day, 20 μL was applied to the corresponding biosensor for AHL quantification.

To assess the effect of YtnP-ZP1 on *P. aeruginosa*-produced long- and short-chain AHLs, bacteria were cultivated for 6 h in the presence or absence (negative control) of the enzyme. After centrifugation, supernatants containing AHLs were filtered and 20 μL of the filtrate were used for quantification of AHL levels using an assay with biosensors.

### Characterization of YtnP-ZP1

All reactions with YtnP-ZP1 were carried out using 10 μg mL^–1^ of the purified enzyme. Characterization of the enzyme was performed according to previously published protocols (Dong et al., 2018) with some modifications. The activity of YtnP-ZP1 was tested in 20 mM sodium phosphate buffer pH 7.4 with 5 μM 3oxo-C12-HSL as a substrate (total reaction volume 1 mL). The reaction mixture was incubated at 30°C (if not stated otherwise) and the catalytic activity of the enzyme was quantified in assays with biosensors. The assays were performed in triplicate and repeated at least three times. The effect of pH on lactonase activity of YtnP-ZP1 was determined within a pH range from 6.0 to 8.0, in 0.5 increments, in 20 mM sodium phosphate buffer (pH 6.0–7.0) and 20 mM Tris–HCl (pH 7.0–8.0) at 30°C. Temperature optimum was determined by performing the enzymatic reactions at 4, 25, 30, 37, and 42°C for 20 h. The thermal stability of the purified enzyme was determined by measuring the residual activity upon incubating the purified enzyme for 2 h at temperatures ranging from 30 to 80°C followed by the enzymatic reactions carried out at 30°C. The effect of various metal ions on YtnP-ZP1 activity was monitored by adding 1mM MgCl_2_, MnSO_4_, FeSO_4_, FeCl_3_, or ZnSO_4_ to the reaction mixture. To determine enzyme kinetics, reactions were carried out in 20 mM sodium phosphate buffer for 20 h at 30°C and the activities were assessed every 2 h.

### Antimicrobial Susceptibility Tests for Planktonic Cells

The minimal inhibitory concentration (MIC) and the minimal bactericidal concentration (MBC) of YtnP-ZP1 and selected antibiotics (tobramycin, gentamicin, and ceftazidime, all purchased from Sigma Aldrich, St. Louis, MO, United States) were determined using standard broth microdilution assays recommended by the Clinical and Laboratory Standards Institute [CLSI] (2012). The stock solutions of antibiotics (10 mg mL^–1^) were prepared in sterile deionized water prior to the experiments. The inoculums were 5 × 10^4^ colony forming units (CFU) mL^–1^. The MIC values corresponded to the lowest concentration that inhibited the visual growth, while MBC values were determined as the lowest concentrations required to kill bacteria after 20 h at 37°C.

To address the effect of combination treatment with lactonase and antibiotics against *P. aeruginosa* PAO1, twofold serial dilutions of tobramycin, gentamicin, or ceftazidime (starting at 32 μg mL^–1^) from horizontal rows of a 96-well microtiter plate were combined with YtnP-ZP1 (10 and 50 μg mL^–1^) and inoculated with bacterial suspension at the final density of 5 × 10^4^ CFU mL^–1^. The MIC and MBC values were determined after 20 h of incubation at 37°C. The assays were carried out in triplicate and repeated twice.

### HPLC Analysis of Acylated Homoserine Lactone Hydrolysis

The hydrolysis of AHLs (3-oxo-C12-HSL and C4-HSL) with YtnP-ZP1 was investigated by High Performance Liquid Chromatography (HPLC) according to published protocol with some modifications (Teiber and Draganov, 2011). AHL stock solutions prepared in methanol (5 mM) were diluted in sodium phosphate buffer (pH 7.4) to final concentrations of 500 μM and then incubated with the enzyme (10 μg mL^–1^, final concentration) at 30°C for 20 h. Control hydrolysis reaction contained NaOH (20 mM) instead of lactonase, and after 20 min incubation at room temperature, the reaction was quenched with HCl (40 mM). Control reactions with 500 μM AHLs and without enzyme were also monitored for 20 h. The optimal concentrations ratio of YtnP-ZP1 and AHLs was determined empirically.

Hydrolysis of AHLs with YtnP-ZP1 or NaOH was monitored by RP-HPLC using the Agilent 1260 HPLC system [Quat Pump (G1311B), Injector (G1329B) 1260 ALS, Thermostated column compartment TCC 1260 (G1316A) and Detector 1260 DAD VL + (G1315C)] with Zorbax Eclipse Plus C18 column [4.6 × 100 mm, 1.8 μm (Agilent, Santa Clara, CA, United States)] as the stationary phase. Mobile phase components [0.1% water solution of HCOOH (A), and acetonitrile (B)] were adjusted for each AHL and the corresponding product of hydrolysis. The analyses were performed at λ = 205 nm. The injection volume was 50 μL, while the flow rate was 0.5 mL min^–1^ and the column temperature was 30°C.

Mobile phase protocols: (i) *C4-HSL*: 90% of solution (A) and 10% of (B). The retention times (t_*R*_) of C4-HSL and the hydrolyzed product butyryl-L-homoserine-*S*-homoserine were 5.92 and 3.80 min, respectively. (ii) *3-oxo-C12-HSL*: 0–4 min 65% A, 4–8 min 65% A → 5% A, 8–9 min 5% A → 65% A, 9–15 min 65% A. The retention times (t_*R*_) of 3-oxo-C12-HSL and the hydrolyzed product N-(3-oxododecanoyl)-*S*-homoserine were 11.20 and 10.02 min, respectively.

### Motility Assays

For motility assays, diluted overnight *P. aeruginosa* PAO1 cultures (1.5–2 × 10^8^ cells mL^–1^) were used. A swarming assay was performed on M8 plates containing 0.6% agar and 50 μg mL^–1^ YtnP-ZP1. Plates were inoculated with 2.5 μL of diluted *P. aeruginosa* PAO1 culture, incubated for 20 h at 37°C, and the size of colony migration was evaluated by measuring the diameter of the covered area (Ha et al., 2014). In the twitching motility assay, diluted overnight *P. aeruginosa* PAO1 cultures were stabbed with a toothpick into LB plates with 1% agar and 50 μg mL^–1^ YtnP-ZP1. The plates were incubated for 72 h at 25°C. Bacterial migration along the plastic surface was detected by crystal violet (2%) staining after the removal of the agar from the Petri dish (Olejnickova et al., 2014). In both motility assays, appropriate plates without YtnP-ZP1 were used as controls.

### Pyocyanin Assay

*Pseudomonas aeruginosa* PA14 strain was cultured overnight in the presence of YtnP-ZP1 at different concentrations (from 10 to 100 μg mL^–1^), and pyocyanin in the supernatant was quantified using UV–Vs spectrophotometry (Ultrospec 3300pro, Amersham Biosciences, Little Chalfont, United Kingdom) at 695 nm, as reported previously (O’Loughlin et al., 2013). The production of pyocyanin was normalized per cell density (*OD*_600_). Experiments were performed in triplicate and repeated three times.

### Elastase Assay

*Pseudomonas aeruginosa* PAO1 cultures were incubated with 50 μg mL^–1^ YtnP-ZP1 for 20 h at 37°C. The elastase activity in the culture supernatants was determined using the Elastin Congo Red (ECR) method (Ohman et al., 1980). A total of 100 μL aliquots of the supernatant was added to 900 μL of ECR buffer containing 5 mg of ECR (Sigma-Aldrich, St. Louis, MO, United States). The mixture was incubated in a rotary shaker for 18 h at 37°C. Insoluble ECR was removed and the A_495 *nm*_ was measured. The elastase activity in the supernatant of treated cultures was normalized per cell density (*OD*_600_) and presented relative to non-treated control (%). Experiments were performed in triplicate and repeated three times.

### Assessment of Anti-biofilm Activity of YtnP-ZP1

#### Static Biofilm Formation Assay

Quantification of biofilm was performed in 96-well microtiter plates using a crystal violet (CV) method for staining the adherent cells (Merritt et al., 2005). Overnight bacterial cultures were diluted to 5 × 10^7^ cells mL^–1^ in LB, and 100 μL was added to the wells without (negative control) or in the presence of YtnP-ZP1. After 24 h incubation at 37°C, biofilms were washed and stained with 0.1% CV (v/v). Assays were performed in six wells and repeated three times.

#### Static Mature Biofilm Disruption Assay

Overnight cultures of bacteria were diluted to 5 × 10^7^ cells mL^–1^ in LB and 100 μL was added to the wells in 96-well microtiter plates and incubated for 24 h at 37 °C. After the removal of non-adherent cells and two washing steps with PBS, the biofilms were treated with different concentrations of YtnP-ZP1 for additional 24 h. Biofilm biomass was quantified using the CV method as described above. Assays were performed in hexaplicate and repeated three times.

### Animal Study in the Zebrafish (*Danio rerio*) Model

Wild type (AB) and transgenic *Tg*(*-2.8fabp10a*:EGFP) zebrafish (*Danio rerio*) embryos, kindly provided by Dr. Ana Cvejić (Wellcome Trust Sanger Institute, United Kingdom), were raised to adult stage in a temperature- and light-controlled zebrafish facility at 28°C and standard 14:10-h light–dark photoperiod. Adult fish were regularly fed with commercial dry food (Special Diet Services, Witham, United Kingdom and TetraMinTM flakes, Tetra Melle, Melle, Germany) twice a day and with *Artemia nauplii* daily. All experiments involving zebrafish were performed in compliance with the European directive 2010/63/EU and the ethical guidelines of the Guide for Care and Use of Laboratory Animals of the Institute of Molecular Genetics and Genetic Engineering, University of Belgrade.

#### Acute and Developmental Toxicity Assessment

Toxicity evaluation in the zebrafish model was carried out following the general rules of the OECD Guidelines for the Testing of Chemicals (OECD Guidelines for the Testing of Chemicals, 2013) and procedures described in literature (Selderslaghs et al., 2009). Briefly, AB zebrafish produced by pair-wise mating were collected, washed, distributed in 24-well plates containing 10 embryos per well and 1 mL of E3 medium (5 mM NaCl, 0.17 mM KCl, 0.33 mM CaCl_2_, and 0.33 mM MgSO_4_ × 7H_2_O), and raised at 28°C. For assessing acute (lethality) and developmental (teratogenicity) toxicity, the embryos at 6 h post-fertilization (hpf) stage were treated with five different concentrations of YtnP-ZP1, antibiotics, and their combination. Embryo water was used as the negative control. Treated embryos were inspected for 22 toxicological parameters (Supplementary Table 2) every day up to 120 hpf under stereomicroscope (Carl Zeiss™ Stemi 508 doc Stereomicroscope, Zeiss, Oberkochen, Germany). Dead embryos were discarded every 24 h. The experiment was performed three times using 20 embryos per concentration. At 120 hpf, embryos were anesthetized by the addition of 0.1% (w/v) tricaine solution (Sigma-Aldrich, St. Louis, MO, United States), photographed, and killed by freezing at −20°C for ≥24 h.

#### Hepatotoxicity Evaluation

The hepatotoxicity was determined using transgenic *Tg*(*-2.8fabp10a*:EGFP) zebrafish embryos with fluorescently labeled liver cells (Her et al., 2003). Zebrafish embryos were exposed to different concentrations of YtnP-ZP1 (25, 50, and 100 μg mL^–1^), tobramycin (MIC and 2 × MIC), and their combination (MIC of tobramycin and 25, 50, or 100 μg mL^–1^ of YtnP-ZP1). Embryos were treated from the 72-hpf stage (when the liver is fully functional, vascularized, and started metabolic transformation of absorbed compounds) to 120-hpf stage, and the hepatotoxicity was assessed by fluorescent microscopy. The liver toxicity was evaluated in relation to the control group according to the following monitored parameters: (i) liver fluorescence and size (decreased size and hepatomegaly), (ii) liver necrosis, (iii) yolk resorption, and (iv) liver area index (the ration between liver area and embryonic lateral area), as predictive parameters of the hepatotoxicity described in the literature (He et al., 2013; Zhang et al., 2017). The experiment was performed two times using 10 embryos per concentration. The liver area index was determined by ImageJ program (NIH, LOCI-UW, United States).

#### Systemic *Pseudomonas aeruginosa*-Zebrafish Infection

##### Pseudomonas aeruginosa PAO1 Culture and Preparation of the Cells for Microinjection

The overnight culture of GFP-expressing *P. aeruginosa* PAO1 (*P. aeruginosa* PAO1 containing pBBR2-GFP) grown in LB broth was diluted at 1:500 ratio and incubated at 37°C with shaking to reach a mid-exponential phase (*OD*_600_ = 0.6–0.7). After centrifugation at 6,000 × *g* for 5 min, the bacterial pellet was washed three times with PBS and resuspended in 2% polyvinyl–pyrrolidone (PVP) to achieve a final concentration of 7–8 × 10^8^ cells mL^–1^.

##### Infection of Zebrafish Embryos

Prior to infection, embryos were manually dechorionated at 24-hpf stage and kept at 28°C. Aiming to establish a fast systemic infection (Milivojevic et al., 2018), 24-hpf dechorionated embryos were anesthetized with 150 μg mL^–1^ of tricaine (MS-222) solution and microinjected with ∼5 nL containing 600–700 *P. aeruginosa* PAO1-GFP cells into the circulation valley by a pneumatic picopump (PV820, World Precision Instruments, Sarasota, FL, United States). The number of injected viable bacteria was determined by counting bacteria from a drop cultured on LB agar plates at 37°C for 1 day. Injected embryos were allowed to recover for 1.5 h at 28°C, dead embryos were discarded, and then live embryos were transferred to 24-well plates containing 1 mL of E3 medium and 10 embryos per well. The infected embryos were treated with the different concentrations of YtnP-ZP1, antibiotics, and their combinations, and maintained at 30°C by 120 hpf. The embryos injected with 2% PVP were used as the control groups. Twenty embryos were used per concentration, and the experiment was performed twice. The survival and development of *P. aeruginosa* PAO1-infected embryos were recorded daily until 120 hpf (corresponding to fourth day post-infection). Antibacterial efficacy of applied treatments was determined according to the survival rate of treated embryos in relation to those in the untreated group.

#### Tail Wound Infection With *Pseudomonas aeruginosa* PAO1

Tail wound infection with *P. aeruginosa* PAO1-GFP was established according to the published protocol (Nogaret et al., 2021) with some modifications. Briefly, to injure the tail fin before infection, 72-hpf old anesthetized AB zebrafish embryos were placed on a 2% agarose plate with tricaine (200 μg mL^–1^), and a small transection of the tail was performed using a 26-gage needle under a stereomicroscope (Carl Zeiss™ Stemi 508 doc Stereomicroscope, Zeiss, Oberkochen, Germany). Injured embryos were immediately immersed in 1 mL of *P. aeruginosa* PAO1-GFP suspension (4–6 × 10^7^ cells mL^–1^ in Embryo water) in 24-well plates and incubated at 28°C until 120 hpf. To evaluate the anti-biofilm efficacy, the wounded embryos were treated with YtnP-ZP1, tobramycin, and their combination just post-immersion into bacterial suspension. The wounded and non-infected embryos were used as the negative control group. Two days post-infection (treatments), the embryos were rinsed two times with embryo water to remove planktonic bacteria, anesthetized, and the tails of wounded embryos were analyzed by fluorescence microscopy.

### Statistical Analysis

GraphPad Prism 6 was used for both statistical analysis and graphical display of the results (GraphPad Software, San Diego, CA, United States). The normality of the distribution was verified in the first phase (Shapiro–Wilk technique). In the instance of the normal distribution, the one-way analysis of variance (ANOVA) test was used, followed by the Fisher’s least significant difference test (LSD). In the case of a non-normal distribution, the non-parametric Kruskal–Wallis test was used, followed by the Dunn’s *post hoc* test. Comparisons in the survival of infected embryos between untreated and treated groups were made using the log-rank test. The Student’s *t*-test was used to perform the statistical analyses for lactonase’s *in vitro* anti-virulence activity assessment using SPSS version 20 software (IBM, Armonk, NY, United States). The significance level was set at *P* < 0.05.

## Results

### Isolation and Characterization of Acylated Homoserine Lactone-Degrading Strain ZP1

**FIGURE 1:**
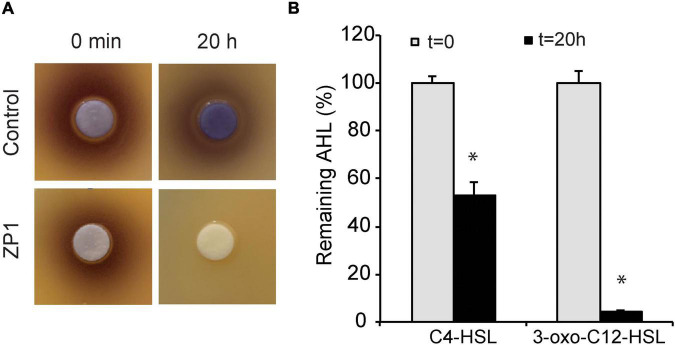
Determination of lactonase activity. **(A)** Hydrolysis of C6-HSL. **(B)** Hydrolysis of C4-HSL and 3-oxo-C12-HSL. Values are plotted relative to buffer-treated control and are representative of two independent experiments ± SD. *P < 0.05.

The AHL-degrading bacterial strain termed ZP1 has been isolated from a sample of agricultural soil through a series of enrichment subculturing in a minimal medium containing C8-HSL. The AHL-degrading capacity of ZP1 was tested after 20 h incubation with C4-, C6-, or 3-oxo-C12-HSL using biosensor strains specific to AHLs with different acyl chain lengths. Biosensor strains used in this study do not produce endogenous AIs but can respond to exogenously added AHLs either by producing pigment violacein in the case of *C. violaceum* CV026 (McClean et al., 1997) or luminescence in the case of *P. aeruginosa* PA14-R3 and PAOJP2 (Duan and Surette, 2007; Massai et al., 2011). The amount of violacein produced or emitted bioluminescence correlated with the concentration of added AHLs. Complete degradation of C6- and 3-oxo-C12-HSL, and the hydrolysis of 50% of C4-HSL (Figures 1A,B) demonstrated higher specificity of ZP1 isolate toward medium- and long- chain AHLs. Genomic DNA of ZP1 was sequenced and the strain was identified as *Bacillus paralicheniformis* with a genome identity of 82.12%. The draft genome and 16S rDNA sequence were deposited in the GeneBank under accession numbers JALIZJ000000000 and ON231782, respectively.

### Heterologous Expression of YtnP Lactonase From *Bacillus paralicheniformis* ZP1 and Its Characterization

Analysis of the annotated *B. paralicheniformis* ZP1 genome revealed the presence of putative QQ lactonase gene *ytnP.* The sequence is deposited in the GeneBank under accession number OM486946. The His-tagged YtnP was overexpressed in *E. coli* and purified by routine metal affinity chromatography procedure. The SDS-PAGE analysis indicated high purity of the obtained recombinant YtnP-ZP1, ∼98% based on the protein staining with Coomassie Brilliant Blue R-250, which can detect as little as 0.1 μg of protein (Supplementary Figure 1). The size of purified YtnP-ZP1 was ∼33 kDa.

**FIGURE 2:**
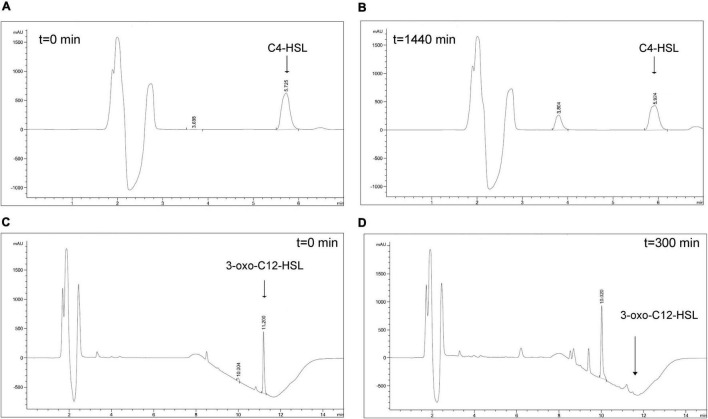
HPLC analysis of AHLs degradation by YtnP-ZP1. Main ion peaks of AHLs standards (*t* = 0) **(A,C**), as well as ring-opened products of AHLs **(B,D)** are presented.

The catalytic activity of purified YtnP-ZP1 was tested using short- and long-chain AHLs (C4- and 3-oxo-C12-HSL) as substrates. The hydrolysis of C4- and 3-oxo-C12-HSL was monitored using HPLC (Figure 2). Untreated AHLs incubated under the same conditions and subsequently subjected to HPLC analysis were used for comparison (Supplementary Tables 3–6). After 24 h incubation with 10 μg mL^–1^ of YtnP-ZP1, 31.8% of C4-HSL remained uncleaved (Figures 2A,B and Supplementary Table 4). However, a similar amount of C4-HSL was also detected in the absence of the enzyme, suggesting its spontaneous degradation under these experimental conditions. YtnP-ZP1 was more effective in hydrolyzing 3-oxo-C12-HSL since it was completely cleaved after 6 h of incubation. No spontaneous degradation of this substrate was observed during the same time period (Figures 2C,D and Supplementary Table 6).

**FIGURE 3:**
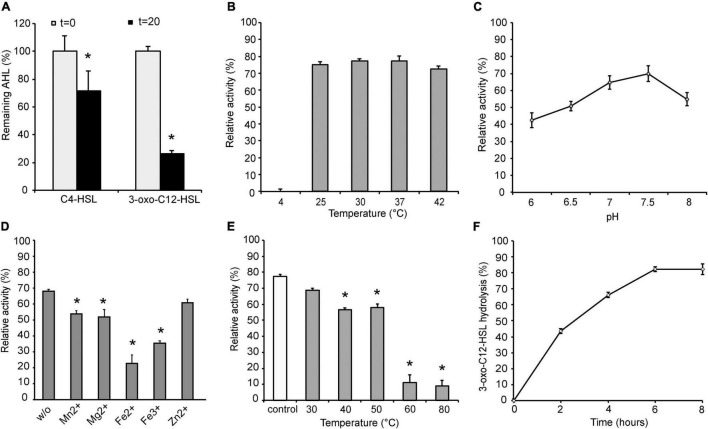
Characterization of YtnP-ZP1. Enzyme specificity **(A)** and the effects of temperature **(B)**, pH **(C)**, metal ions **(D)**, pre-incubation temperature **(E)** on lactonase activity and the rate of 3-oxo-C12-HSL hydrolysis **(F)**. w/o, control reaction without metal ions; control, reaction with enzyme without pre-incubation. Values are representative of three independent experiments ± SD. *P < 0.05.

The activity of purified YtnP-ZP1 was additionally evaluated in the assays with biosensors. The obtained results confirmed higher YtnP-ZP1 activity against long-chain AHL since 75% of 3-oxo-C12-HSL has been hydrolyzed in comparison with 30% of hydrolyzed C4-HSL (Figure 3A). All subsequent characteristics of purified YtnP-ZP1 were determined on its preferential substrate 3-oxo-C12-HSL.

The temperature optimum of YtnP-ZP1 at neutral pH 7.4 was determined to be in the range between 25 and 42°C at which the enzyme retained at least 80% of substrate hydrolysis rate (Figure 3B). The pH optimum determined at 30°C in the pH range of 6.0–8.0 was 7.5, at which maximum enzyme activity was obtained after 20 h incubation (Figure 3C).

Metal ions such as Mn^2+^, Mg^2+^, and Zn^2+^ slightly decreased YtnP-ZP1 activity at 1 mM concentration, while in the presence of Fe^2+^ and Fe^3+^ the lactonase activity was reduced by 45 and 33%, respectively (Figure 3D).

To address the thermostability of YtnP-ZP1, the enzyme was pre-incubated at different temperatures for 2 h before providing the substrate. The enzyme proved to be stable when pre-incubated at temperatures up to 50°C, losing 20% of its activity (Figure 3E). However, a further increase of pre-incubation temperature led to complete enzyme inactivation.

Finally, the reaction rate of 3-oxo-C12-HSL hydrolysis with YtnP-ZP1 was monitored at previously established optimal conditions, and the remaining substrate concentration was measured every 2 h using the biosensor. The reaction maximum with 80% of 3-oxo-C12-HSL hydrolysis was reached after 6 h (Figure 3F).

### Effect of YtnP-ZP1 on Quorum Sensing in *Pseudomonas aeruginosa*

**FIGURE 4:**
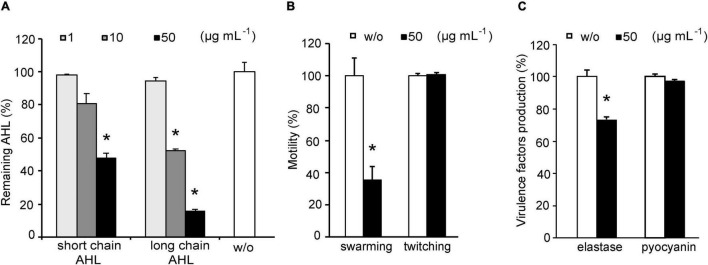
Effects of YtnP-ZP1 on *P. aeruginosa* PAO1 lactones **(A)**, motility **(B)**, and on elastase and pyocyanin production **(C)**. Values are plotted relative to buffer-treated control (w/o) and are representative of three independent experiments ± SD. *P < 0.05.

Pathogenicity of *P. aeruginosa* highly depends on the virulence characteristics regulated by AHLs. Thus to evaluate anti-virulence potential of YtnP-ZP1, we tested whether it can hydrolyze AHLs in *P. aeruginosa* culture. Overnight bacterial cultures were subcultured with the different concentrations of the lactonase for 6 h and the relative amounts of residual AHLs in the supernatants were determined using specific *P. aeruginosa* biosensors. Consistent with the results obtained with commercial AHLs (Figure 3A), YtnP-ZP1 appeared to be more effective in hydrolyzing long-chain than short-chain AHLs showing 85 and 50% reduction of AHLs in the supernatant, respectively, at 50 μg mL^–1^ (Figure 4A). Further increase in the lactonase concentration did not change its efficacy (data not shown).

The effects of YtnP-ZP1 on AHL-regulated virulence characteristics in *P. aeruginosa* were assessed by measuring the level of motility, elastase, and pyocyanin production after exposing the bacteria overnight to 50 μg mL^–1^ of the enzyme. Swarming motility was reduced by 60%, while no effect on twitching was observed (Figure 4B and Supplementary Figure 2). Incubation with YtnP-ZP1 led to reduced elastase activity in *P. aeruginosa* supernatants by 25%, but it did not affect the production of pyocyanin (Figure 4C). Importantly, YtnP-ZP1 in tested concentrations did not affect bacterial growth during incubation for 24 h (Supplementary Figure 3), suggesting that the observed effects could be ascribed to its QQ activity.

**FIGURE 5:**
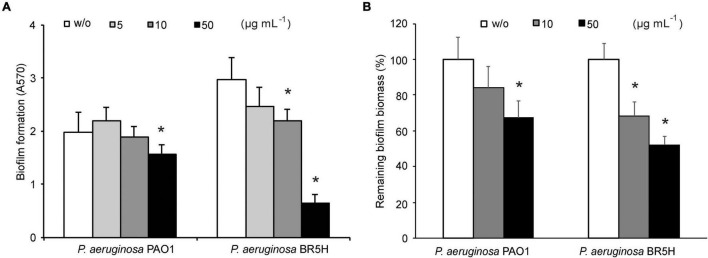
Effects of YtnP-ZP1 on *P. aeruginosa* PAO1 biofilms. Biofilm formation in the absence (w/o) or presence of lactonase **(A)**. Inhibition of *P. aeruginosa* mature biofilms. Values are plotted relative to buffer-treated control (w/o) **(B)**. Values are representative of three independent experiments ± SD. *P < 0.05.

Since biofilm formation contributes substantially to the virulence of *P. aeruginosa* and is quite often responsible for diminished therapeutic efficacy of applied antibiotics, we have evaluated the effect of YtnP-ZP1 on *P. aeruginosa* biofilms. We found that YtnP-ZP1 reduced biofilm formation in *P. aeruginosa* PAO1 by 25% at the highest applied dose of 50 μg mL^–1^ (Figure 5A). Notably, this effect was more prominent in multiresistant *P. aeruginosa* clinical strain BR5H isolated from a patient with an infected wound (Milivojevic et al., 2018), where the treatment with the same concentrations of YtnP-ZP1 inhibited 85% of biofilm formation. Exposing the mature *P. aeruginosa* biofilms to increasing concentrations of YtnP-ZP1 led to a reduction of biofilm biomass by 35 and 50% in PAO1 and BR5H strains, respectively (Figure 5B).

### Biofilm Inhibition in Environmental Opportunistic Pathogens

**FIGURE 6:**
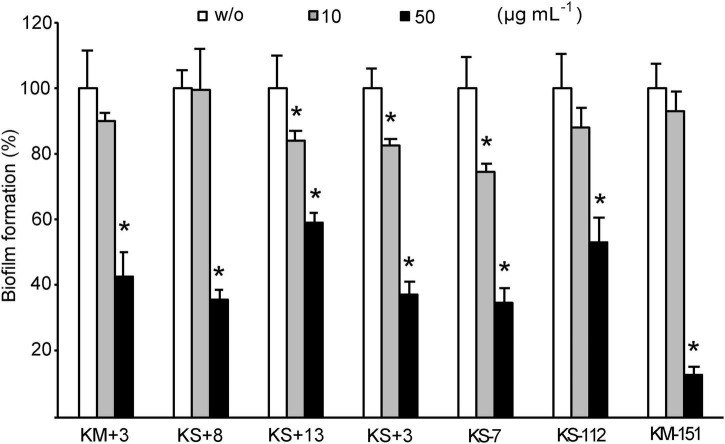
Effects of YtnP-ZP1 on biofilm formation in environmental isolates. Values are plotted relative to buffer-treated control (w/o) and are representative of three independent experiments ± SD. *P < 0.05.

The versatility of biofilm inhibition activity of YtnP-ZP1 was tested on 18 best biofilm-producing bacterial strains from the environmental in-house ZP collection (Supplementary Table 7). YtnP-ZP1 showed anti-biofilm activity against seven isolates with biofilm reduction ranging from 40 to 90% (Figure 6). The lactonase in applied concentrations did not affect bacterial growth and did not reduce the biomass of mature biofilms in any of the screened isolates (data not shown).

The 16S rDNA sequence analysis revealed that all seven isolates belonged to the Gram-negative opportunistic pathogens, most of which were able to cause infection in humans. Two isolates belonged to *Pseudomonas* species, two to *Brucella/Ochrobactrum* species, one to *Agrobacterium*, one to *Sphingobacterium*, and one to *Serratia* species (Table 1). Phenotypic characteristics of biofilm-forming isolates are presented in Supplementary Table 8.

**TABLE 1:** Identification of environmental isolates selected based on the level of biofilm inhibition in the presence of YtnP-ZP1.

Isolate	16S rDNA similarity		GeneBank Acc. no.
	Genus	Closest species	
KM + 3	*Brucella/Ochrobactrum*	*Brucella anthropi/Ochrobactrum anthropi* (99.93%)	ON306428
KS + 3	*Pseudomonas*	*Pseudomonas extremorientalis* (100%)	ON306427
KS-7	*Sphingobacterium*	*Sphingobacterium multivorum* (99.79%)	ON306429
KS + 8	*Agrobacterium*	*Agrobacterium tumefaciens* (100%)	ON306430
KS + 13	*Brucella/Ochrobactrum*	*Brucella anthropi/Ochrobactrum anthropi* (99.93%)	ON306431
KS-112	*Pseudomonas*	*Pseudomonas lini* (100%)	ON306432
KM-151	*Serratia*	*Serratia liquefaciens* (100%)	ON306433

### Effect of YtnP-ZP1 on Antibiotics Activity *in vitro*

Anti-virulence agents including QQ enzymes are aimed to be used together with antibiotics to increase their efficacy. In treating *P. aeruginosa* infections antibiotics of choice are typically aminoglycosides (tobramycin and gentamicin) and cephalosporines (ceftazidime); thus we examined whether YtnP-ZP1 affects the activity of these antibiotics *in vitro*. YtnP-ZP1 alone showed no effect on *P. aeruginosa* PAO1 growth even at high concentrations up to 200 μg mL^–1^ (Supplementary Figure 3B). When applied together with tobramycin, gentamicin, or ceftazidime, lactonase showed no effect on their activity since their MIC and MBC values remained unchanged (Supplementary Table 9).

### Toxicity Assessment

In order to address whether YtnP-ZP1 is safe for human use, we first evaluated its cytotoxicity toward human cells in culture. In concentrations up to 200 μg mL^–1^, YtnP-ZP1 did not affect the viability of human fibroblasts (MRC5; Supplementary Figure 4).

**FIGURE 7:**
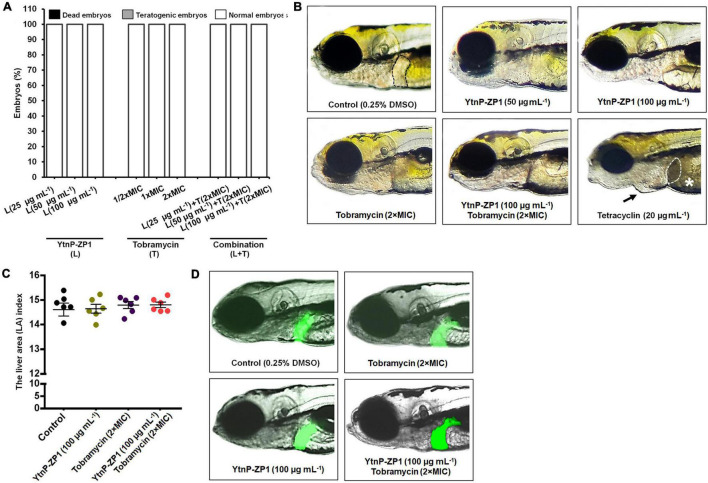
*In vivo* toxicity evaluation of YtnP-ZP1, tobramycin, and their combination in the zebrafish (*Danio rerio*) model. Acute toxicity was assessed using wild-type embryos exposed to the different doses of enzyme, antibiotic, and their combination **(A)**. Embryos were treated at 6 hpf and evaluated for survival, teratogenicity, cardiotoxicity, and hepatotoxicity at 120 hpf (*n* = 60 per a dose). Normally developed embryos, without signs of hepato- and cardiotoxicity, after exposure to 100 μg mL^–1^ of YtnP-ZP1, 2 × MIC of tobramycin and the combination of 100 μg mL^–1^ lactonase and 2 × MIC tobramycin are shown **(B)**. The normal (clear) liver in a control embryo is outlined. Tetracycline caused liver necrosis and darkening (dashed outlined), yolk necrosis (asterisk), and pericardial edema (arrow). The hepatotoxicity of the tested enzyme and its combination with antibiotic was additionally verified in zebrafish embryos with fluorescently labeled liver (*n* = 6) according to the liver fluorescence **(C)** and the liver area index **(D)**.

Zebrafish (*D. rerio*) embryos are a well-established preclinical animal model system; thus we used it to examine acute and inner organs toxicity of YtnP-ZP1 treatments. As shown in Figure 7, the enzyme applied in up to 100 μg mL^–1^ dose did not cause any visible toxic side effect, including the signs of cardiotoxicity (pericardial edema), hepatotoxicity (liver necrosis and reduced yolk absorption), and teratogenicity (body malformations).

Likewise, no side effects had been detected when YtnP-ZP1 was combined with antibiotics tobramycin (Figure 7), gentamicin, or ceftazidime (Supplementary Figure 5). As a positive control, we used the antibiotic tetracycline, known to cause the pronounced pericardial edema and liver necrosis (darkening) (Sovari et al., 2020).

In order to exclude the harmful effects of YtnP-ZP1 on liver, the transgenic *Tg(-2.8fabp10a:EGFP)* zebrafish embryos with fluorescently labeled liver were exposed to YtnP-ZP1 lactonase (100 μg mL^–1^), tobramycin (2 × MIC), and their combination. The treated embryos were examined by fluorescence microscopy for the liver size and fluorescence and analyzed for the liver area index as an important hepatotoxicity endpoint. Data obtained in this assay did not show the difference in liver area index between the control (DMSO-treated) group and groups treated with YtnP-ZP1, tobramycin, and their combination (*P* > 0.05). Taken together, these data indicate that YtnP-ZP1, applied in bioactive concentrations alone or in combination with tobramycin, does not produce toxic side effects, including cardio- and liver toxicity.

### Treatments of Systemic *Pseudomonas aeruginosa* PAO1 Infection *in vivo*

After demonstrating anti-biofilm and anti-virulence activity of YtnP-ZP1 *in vitro*, we investigated the enzyme’s therapeutic potential against *P. aeruginosa* infections *in vivo* using the zebrafish model of systemic and wound infections.

**FIGURE 8:**
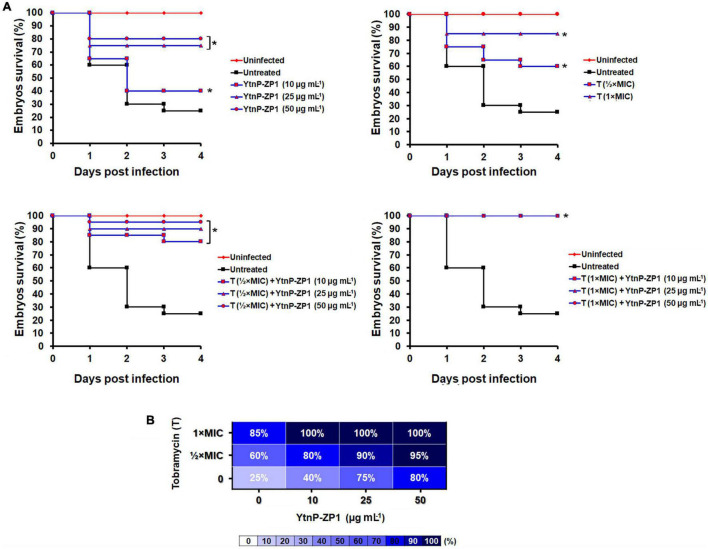
YtnP-ZP1 efficiently rescued the zebrafish embryos of the lethal *P. aeruginosa* PAO1-GFP-infection. Wild-type zebrafish embryos were infected with *P. aeruginosa* PAO1-GFP in the circulation valley and incubated at 32°C (*n* = 20 per dose). The Kaplan–Meier survival curves **(A)** and the survival heat map at 120 hpf **(B)** of *P. aeruginosa* PAO1-infected embryos upon the different treatments are shown. *P < 0.05.

First, we employed the zebrafish-*P. aeruginosa* PAO1 model of infection to establish a rapid systemic infection by injecting 600–700 bacterial cells into the bloodstream (the circulation valley) of 28-hpf old embryos. Infected embryos were treated with the different concentrations of YtnP-ZP1, tobramycin, gentamicin, ceftazidime, or their combination and inspected for the survival over a period of 4 days post-infection (dpi) (Figure 8 and Supplementary Figures 6, 7).

Contrary to the survival rate of only 25–30% in the untreated group, YtnP-ZP1 applied at doses ≥ 25 μg mL^–1^, as well as tobramycin at the doses ≤ MIC, significantly improved the survival of *P. aeruginosa* PAO1-infected embryos (*P* < 0.01, log-rank assay). However, none of the applied treatments provided the survival of all infected embryos, rescuing 80 and 85% embryos upon the highest applied dose of YtnP-ZP1 and antibiotics, respectively (Figure 8A, upper panels). Importantly, combination treatment with enzyme and tobramycin at 1/2 × MIC improved the survival rate reaching 100% (Figure 8A, lower panels and Figure 8B). Similarly, the increased efficacy of gentamicin with YtnP-ZP1 was observed (Supplementary Figure 6); however, when applied together with ceftazidime YtnP-ZP1 showed antagonistic activity (Supplementary Figure 7).

### Treatments of Tail Wounds Infected With *Pseudomonas aeruginosa* PAO1

**FIGURE 9:**
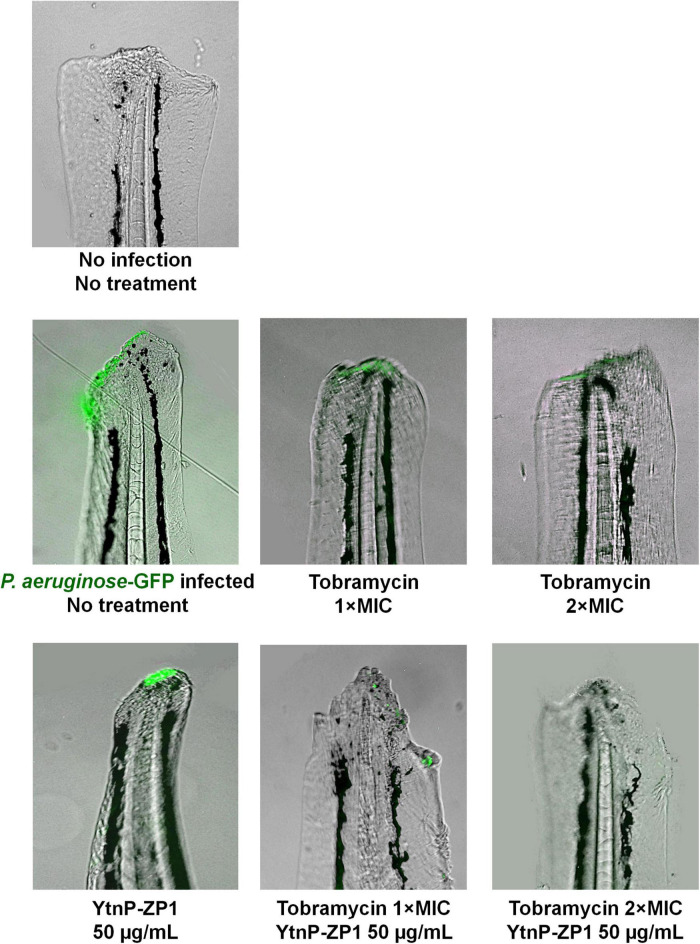
*Pseudomonas aeruginosa* PAO1 tail wound infection was successfully eliminated by applying YtnP-ZP1 in combination with tobramycin. Wild-type zebrafish embryos were decapitated at 72 hpf and infected with 4–6 × 10^7^ cells of *P. aeruginosa* PAO1-GFP. After 1–2 h of recovery, wounded embryos were treated with tobramycin, lactonase, or their combination. Individual treatment with enzyme or antibiotic partially eliminated infection from the wounded tail, while combination treatment with lactonase and tobramycin was more effective resulting in no visible bacterial infection (lack of fluorescence).

Having in mind that *P. aerugin*osa is one of the most common causal agents of the wound infections in humans, we employed the newly established zebrafish tail wound infection model to evaluate anti-virulence and the anti-biofilm efficacy of YtnP-ZP1 *in vivo*. The wounded tails of 72-hpf old embryos were infected with GFP-expressing *P. aerugin*osa and treated with YtnP-ZP1 (50 μg mL^–1^), tobramycin (MIC and 2 × MIC), and their combinations (Figure 9). After 2-days treatment, the infected embryos were first thoroughly washed to remove planktonic bacteria and then analyzed for the biofilm-associated infection at the wounded tissue using fluorescence microscopy. We found that treatment with either YtnP-ZP1 or tobramycin was similarly effective and only partially eliminated the bacterial biofilms from the wounded tissue. The combined treatment of enzyme (50 μg mL^–1^) with antibiotic (2 × MIC) proved to be much more effective resulting in no visible biofilms (observed as the lack of fluorescence).

## Discussion

Inhibition of QS using small molecules or enzymes is considered one of the most promising anti-virulence approaches that enables the inhibition of biofilm formation and limits the pathogenicity of bacteria. Small molecules work by entering the bacterial cells and inactivating target proteins such as AI synthases or receptors, while enzymes act on extracellular targets. Anti-virulence agents do not affect the pathogen growth and thus induce less selective pressure than antibiotics. However, several reports demonstrated that the treatments with small molecule inhibitors of QS led to mutations that increased the activity of efflux pumps causing resistance to the applied treatment (Maeda et al., 2012). Importantly, resistance to QQ enzymes has not been reported so far, most likely due to their activity on extracellular substrates. Other advantages of QQ enzymes over small molecules inhibiting QS are low or no toxicity, minimal side effects, sustainable production, and that they are environmental-friendly.

Numerous efforts have been dedicated to identify QQ enzyme producers in the samples isolated from different environments, aiming to discover enzymes with broad stability and high specificity toward the target of interest. In the search for novel QQ enzymes that could be used in the treatment of infections caused by the most common Gram-negative bacteria, we isolated bacteria from agricultural soil heavily treated with aromatic herbicides based on their ability to grow on AHLs as the sole carbon source. We assumed that long-term exposition to high concentrations of aromatic compounds selected for bacterial strains expressing enzymes highly effective in cyclic molecules hydrolysis. The enzyme responsible for AHL-degrading activity of isolate classified as *Bacillus paralycheniformis* was identified as YtnP lactonase. It contained the common lactonase motif HXHXDH required for AHL hydrolysis (Thomas et al., 2005) and showed 99.29% identity with putative metallo-β-lactamase fold metallohydrolase from *B. paralichenifromis* (WP_145651162). Based on the BLASTp results, these enzymes are highly conserved across different *Bacillus* species showing identity above 75%.

Our study describes the first characterization of YtnP from *B. paralicheniformis* and one of only a few YtnP enzymes from the *Bacillus* genus (Schneider et al., 2012; Rosier et al., 2020; Peng et al., 2021). To the best of our knowledge, there are three reports on the expression and purification of YtnP derived from *Burkholderia cepacia* (Malesevic et al., 2020), *Acinetobacter baumanii* ATCC17978 (Mayer et al., 2018), and *Bacillus licheniformis* (Peng et al., 2021). YtnP-ZP1 showed very low similarity on the protein level with the mentioned YtnP from *B. cepacia* and *A. baumanii*, 21.6 and 20.3%, respectively, while it showed 96% similarity to YtnP from *B. licheniformis.*

Purified YtnP-ZP1 displayed a broad spectrum of activity being able to hydrolyze different AHL synthetic standards. Notably, YtnP-ZP1 was more effective in hydrolyzing 3-oxo-C12-HSL compared to C4-HSL, suggesting its specificity toward long-chain AHLs. The specificity of QQ lactonases is important for their application since enzymes with differential specificity toward AHLs, despite similar anti-virulence activity *in vitro*, demonstrated quite a different efficacy in the *in vivo* model of infection (Remy et al., 2020). Lactonase showing broader activity toward AHLs demonstrated lower efficacy in the *in vivo* model of infection with *P. aeruginosa* PA14 compared to the enzyme specific for long-chain AHLs (Remy et al., 2020).

Lactonases differ in their thermostability, with the ones isolated from thermophilic organisms being more stable. YtnP-ZP1 proved to be thermostable at temperatures up to 50°C with optimal activity at neutral pH, similar to YtnP from *B. cepacia* and other reported lactonases (Chen et al., 2010; Cao et al., 2012; Malesevic et al., 2020). Although several reports stated that lactonases are metalloproteins (Thomas et al., 2005; Momb et al., 2006), YtnP-ZP1 does not require metal ions for its activity. Actually, the enzyme activity was reduced to a different degree in the presence of tested metal ions, with the lowest activity in the presence of iron. These results are in line with the findings of Cao and co-workers who also found that Fe^2+^ partially inhibits AiiA lactonase activity (Cao et al., 2012), while the enzyme was partially inhibited by Cr^2+^ and Pb^2+^, and completely inhibited by Cu^2+^ (Wang et al., 2004) suggesting that the effect of metals ions on the lactonases’ activity depends on the type of the enzyme.

In *P. aeruginosa*, the QS system consists of four interconnected and hierarchically organized signaling pathways (Papenfort and Bassler, 2016). Two of them, Las and Rhl, are regulated by 3-oxo-C12-HSL and C4-HSL, respectively, both of which play an important role in regulating *P. aeruginosa* pathogenicity. The Las signaling pathway regulates the expression of virulence factors such as elastase and alkaline protease which are important for the host’s tissue damage and bacterial invasion during infection, while Rhl regulates the production of rhamnolipids and biofilm formation (Lee and Zhang, 2015). Other virulence traits that contribute to *P. aeruginosa* pathogenicity include three types of motilities, swimming, swarming, and twitching, which are used for the colonization of different environments. Swarming and twitching are AHL-regulated, while swimming is QS-independent (Glessner et al., 1999; Daniels et al., 2004). Reduction of AHL’s level in *P. aeruginosa* supernatants in the presence of YtnP-ZP1 led to reduced elastolytic activity and swarming motility, but did not affect other virulence traits such as pyocyanin production or twitching motility. YtnP-ZP1 treatment inhibited the formation of biofilms, the major virulence determinant of *P. aeruginosa* and many other pathogenic bacteria, which are involved in low sensitivity or resistance to antibiotics (Yan and Bassler, 2019). The enzyme was more effective in inhibiting biofilms from clinical isolate *P. aeruginosa* BR5H, a multiresistant strain isolated from infected wound that forms thicker biofilms, than the reference strain *P. aeruginosa* PAO1 (Milivojevic et al., 2018). In *P. aeruginosa* QS, the network varies between the strains and evolves during the contact with hosts and other bacteria, suggesting that lactonase activity and therapeutic potential should be tested in different strains. Importantly, YtnP-ZP1 was able to inhibit biofilm formation in several other opportunistic pathogens with the highest activity against *Serratia* biofilms. These bacteria use a wide array of AHLs as QS signaling molecules ranging from long-chain in *Pseudomonas* (C12), medium-chain in *Agrobacterium tumefaciens* (C8) (Haudecoeur et al., 2009) to short-chain in *Serratia* (C4, C6) (Van Houdt et al., 2007) demonstrating high versatility of lactonase activity. Most of these bacteria had been previously proven to have LuxI/LuxR type QS system (Rutherford and Bassler, 2012) except for *Sphingobacterium*, where very few data are available. However, genome mining of *Sphingobacterium* sp. S2 showed that it harbors gene homolog to orphan LuxR transcription factor (Satti et al., 2021), and susceptibility to YtnP-ZP1 indirectly points to AHL-mediated QS system.

Although YtnP-ZP1 only partially inhibited *P. aeruginosa* virulence activity in *in vitro* assays, we demonstrated that it effectively eradicates the systematic and local (wound) infections in zebrafish infection models. Zebrafish embryos are powerful and reliable preclinical tools for toxicity and bioactivity evaluation of novel antimicrobial agents (Chakraborty et al., 2009; MacRae and Peterson, 2015; Patton et al., 2021) and are widely used as the animal host for modeling human acute and chronic infections and studying host–pathogen interactions (Clatworthy et al., 2009; Nogaret et al., 2021). Our results showed that YtnP-ZP1 alone or in combination with tobramycin, gentamicin, or ceftazidime is safe, without any visible signs of cardiotoxicity and hepatotoxicity that are common drawbacks of clinically used anti-infectious agents.

Treatment with YtnP-ZP1 effectively rescued zebrafish embryos of lethal *P. aeruginosa* PAO1 systemic infection. When applied with aminoglycoside antibiotics, the treatment appeared more effective than the antibiotic or the enzyme alone, enabling the survival of all infected embryos. We also found that YtnP-ZP1 alone or in combination with tobramycin was effective in preventing *P. aeruginosa* PAO1 wound infection. Although YtnP-ZP1 does not affect pathogen growth, its high efficacy in clearing infections *in vivo* could be explained by the ability to attenuate QS regulated *P. aeruginosa* virulence which makes bacteria more susceptible to the host’s immune system and co-applied antibiotic. Contrary to the combination with aminoglycosides, YtnP-ZP1 showed antagonistic activity toward ceftazidime, which could be explained by the partial inactivation of antibiotic by enzyme through its β-lactamase activity and the possibility that the cleavage product makes either zebrafish embryos more susceptible to *P. aeruginosa* infection or bacteria more virulent. To elucidate how YtnP lactonases affect β-lactam antibiotics efficacy, the observed phenomenon should be further investigated.

A recent report showed that intraperitoneal administration of YtnP lactonase from *B. licheniformis* increased the survival of *Carassius auratus* infected with *Aeromonas hydrophila* proposing its usage as the prophylactic agent in aquaculture practices (Peng et al., 2021). We demonstrated that YtnP-ZP1 could be used as an antibiotic adjuvant increasing its efficacy in the treatment of systemic *P. aeruginosa* infections. In addition, our results suggest that YtnP lactonase could be used as a topical agent on wounded skin to prevent chronic, biofilm-associated wound infections caused by *P. aeruginosa* and other Gram-negative bacteria employing AHL-regulated virulence.

In conclusion, here we report the isolation of *B. paralicheniformis* ZP1 that produces a potent quorum quenching YtnP lactonase with a broad substrate range but with higher affinity toward long-chain AHLs. Such substrate specificity resulted in the reduction of QS-regulated virulence in *P. aeruginosa* and strong inhibition of biofilm formation in different Gram-negative bacteria. The absence of toxicity, high efficacy in eradication of *P. aeruginosa* infections, and the potential to increase the efficacy of aminoglycosides *in vivo* demonstrate that YtnP-ZP1 is a good candidate for developing novel antimicrobial therapeutics. The extracellular mode of activity and no effect on bacterial growth suggest that the resistance to lactonase is unlikely to occur. Thus, lactonases including YtnP-ZP1 should be further examined as the prophylactic agent against different Gram-negative bacterial infections.

## Data Availability Statement

The datasets presented in this study can be found in online repositories. The names of the repository/repositories and accession number(s) can be found below: https://www.ncbi.nlm.nih.gov/bioproject/, PRJNA822920; https://www.ncbi.nlm.nih.gov/biosample, SAMN27282932.

## Ethics Statement

Ethical review and approval was not required for the study because all experiments involving zebrafish were performed in compliance with the European directive 2010/63/EU and the ethical guidelines of the Guide for Care and Use of Laboratory Animals of the Institute of Molecular Genetics and Genetic Engineering, University of Belgrade.

## Author Contributions

LS designed overall research. LD, NS, AP, IM, and LS wrote sections of the manuscript. All authors have made a substantial, direct, and intellectual contribution to the work, and approved the submitted version.

## Conflict of Interest

The authors declare that the research was conducted in the absence of any commercial or financial relationships that could be construed as a potential conflict of interest.

## Publisher’s Note

All claims expressed in this article are solely those of the authors and do not necessarily represent those of their affiliated organizations, or those of the publisher, the editors and the reviewers. Any product that may be evaluated in this article, or claim that may be made by its manufacturer, is not guaranteed or endorsed by the publisher.

## Footnotes

1
http://rast.nmpdr.org
