# Supplementary material for: Novel Quorum Quenching YtnP Lactonase From Bacillus paralicheniformis Reduces Pseudomonas aeruginosa Virulence and Increases Antibiotic Efficacy in vivo
Source: Front Microbiol. 2022 Jun 2;13:906312. doi: 10.3389/fmicb.2022.906312 (PMC9201388; doi:10.3389/fmicb.2022.906312)
Supplement: Supplementary file 1 [file Data_Sheet_1.pdf]

frontiers

Supplementary Material

Novel quorum quenching YtnP lactonase from *Bacillus paralicheniformis* reduces *Pseudomonas aeruginosa* virulence and increases antibiotic efficacy *in vivo*

Lidija Djokic[1], Nada Stankovic[1], Ivana Galic[1], Ivana Moric[1], Natasa Radakovic[1], Sandra Šegan[2], Aleksandar Pavic[1], Lidija Senerovic[1]*

[1] Institute of Molecular Genetics and Genetic Engineering, University of Belgrade, Vojvode Stepe 444a, 11 042, Belgrade 152, Serbia
[2] University of Belgrade, Institute of Chemistry, Technology and Metallurgy, National Institute of the Republic of Serbia, Njegoševa 12, P.O. Box 473, 11000 Belgrade, Serbia

*** Correspondence:**
Lidija Senerovic
seneroviclidija@imgge.bg.ac.rs

1 Supplementary methods

1.1 Phenotypic characterization of environmental isolates

The ability of bacterial isolates for citrate utilization and urea degradation was assayed on Simmons citrate agar (Torlak, Serbia) and Christensen′s urea agar (Torlak, Serbia), respectively according to manufacturers' instructions. Catalase test was done with 3% H_2O_2 according to Bailey & Scott, 2014. Cellulolytic activity was detected on carboxymethyl cellulose plates flooded with iodine solution as described in Kasana et al., 2008. Lipase activity was detected on plates containing Tween 80 according to Plou et al. (1998). DNase activity was detected on DNase test agar plates (HiMedia, India) flooded with HCl, according to manufacturers' instructions. Starch hydrolysis was detected by starch agar protocol (American Society for Microbiology, 2012) on plates flooded with Gram's iodine. Sugar utilization was tested in liquid MSM medium (HiMedia, India) supplemented with 1% sugar (glucose, fructose, saccharose, or lactose) as a sole carbon source, according to manufacturers' instructions. Hemolytic activity was tested on Columbia agar plates supplemented with 5% sheep blood (bioMérieux, France). All chemicals were procured from Merck & Co., Inc. (USA), unless stated otherwise.

1.2 Cytotoxicity assay

MRC-5 human lung fibroblasts were obtained from the American Type Culture Collection. Cells were maintained as monolayer cultures in RPMI-1640 supplemented with 100 mg L^{-1} streptomycin, 100 U mL^{-1} penicillin and 10% (v/v) FBS (all from Sigma, Munich, Germany). Cells were grown in a humidified atmosphere of 95 % air and 5% CO_2 at 37 °C.

Cytotoxicity on MRC-5 cells was evaluated with 3-(4,5-dimethylthiazol-2-yl)-2,5-diphenyltetrazolium bromide (MTT). Assay was carried out after 48 h of cell incubation in the media containing test compounds at different concentrations and the viability was measured as described (Mihajlovic et al., 2012). The results are presented as percentage of the control (untreated cells) that was arbitrarily set to 100 %.

2 Supplementary Figures and Tables

2.1 Supplementary Figures

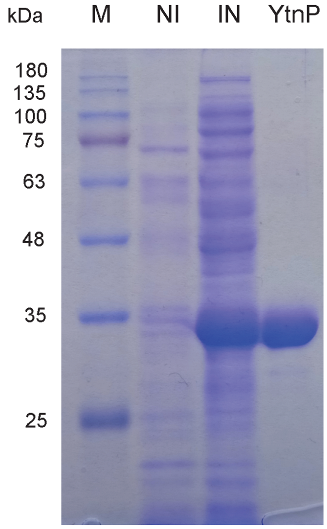

Supplementary Figure 1. SDS-PAGE of purified YtnP-ZP1. M - Blue Star protein marker, NI - non-induced culture, IN - induced culture, YtnP - purified enzyme.

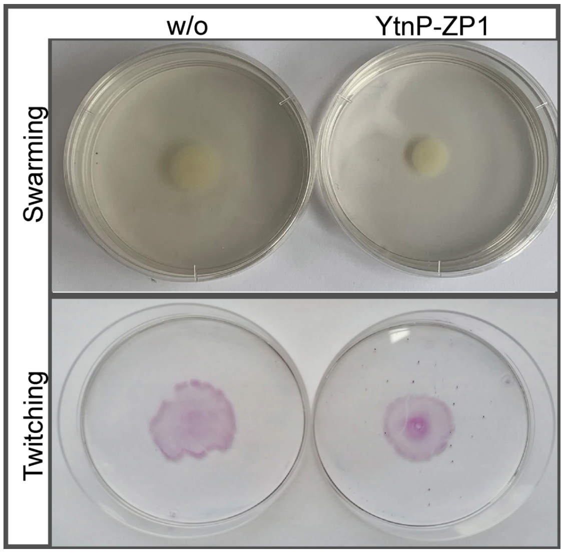

Supplementary Figure 2: Representative images of the effects of YtnP-ZP1 (50 µg mL^{-1}) on *P. aeruginosa* PAO1 motility. w/o represents motility in the absence of enzyme.

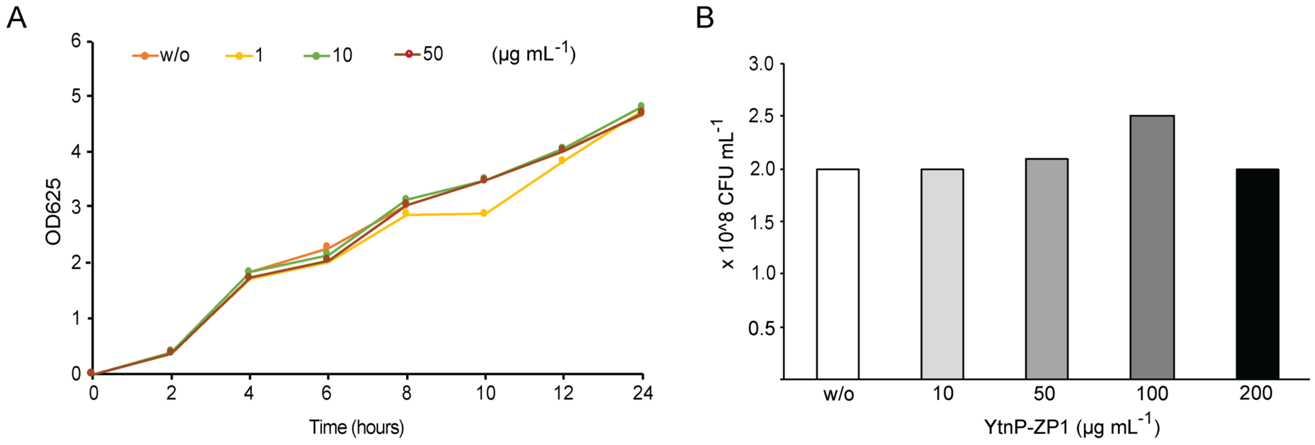

Supplementary Figure 3. Effect of YtnP-ZP1 on bacterial growth. Optical density of *P. aeruginosa* PAO1 cultures grown in the absence (w/o) or presence of increasing concentrations of YtnP-ZP1 (A). Bacterial numbers (CFU mL^{-1}) after incubation with or without (w/o) enzyme for 24 h (B).

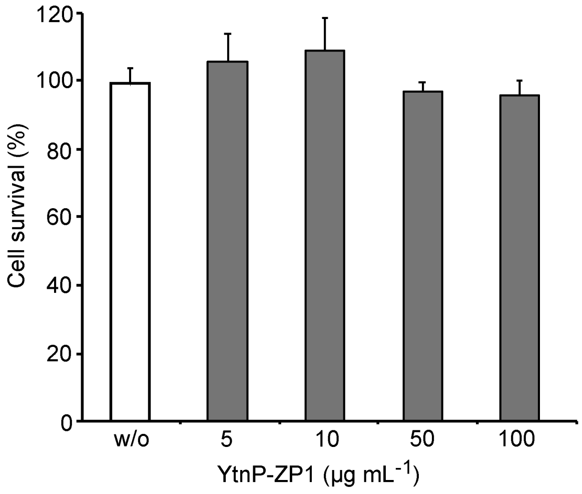

Supplementary Figure 4. Cytotoxicity of YtnP-ZP1 to human lung fibroblast (MRC-5) cell line after 48 h exposure at a range of concentrations. Viability of cells without treatment was labeled as w/o. All values are plotted relative to buffer-treated control and presented as mean ± SD.

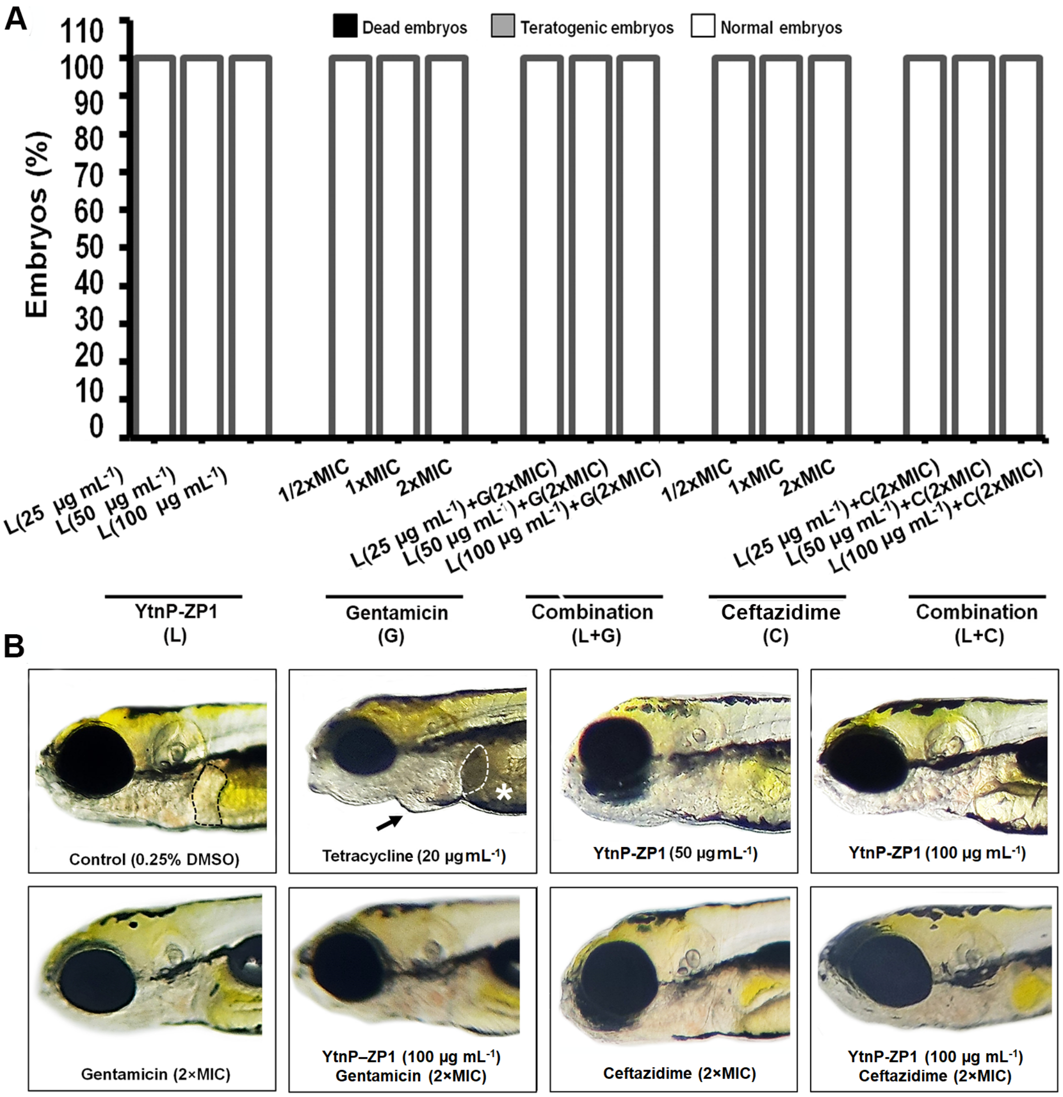

Supplementary Figure 5. *In vivo* toxicity of YtnP-ZP1, gentamicin and ceftazidime, and their combinations assessed in the zebrafish (*Danio rerio*) model. The dose-dependent effect of applied treatments on the survival and teratogenicity (A), as well as morphology of zebrafish embryos (B) are shown. Tetracycline was used as a hepatotoxic antibiotic that caused the liver necrosis (dashed outlined area), yolk necrosis (asterisk) and an appearance of pericardial edema (arrow).

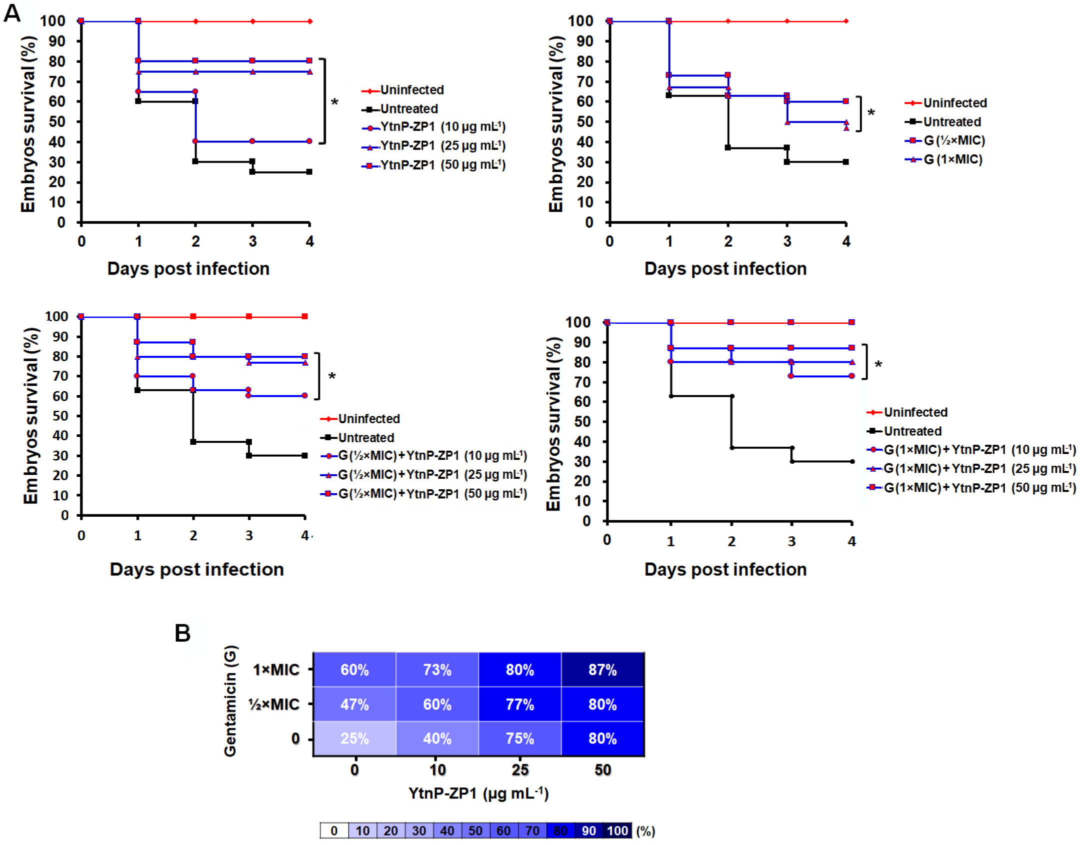

Supplementary Figure 6. Antibacterial efficacy of YtnP-ZP1 (L), gentamicin (G) and their combinations was assessed in the *P. aeruginosa* PAO1-zebrafish infection model. The Kaplan-Meier survival curves of the untreated and treated infected embryos during four days post infection (A) as well as the cumulative survival rate at the end of the experiment (B) are shown.

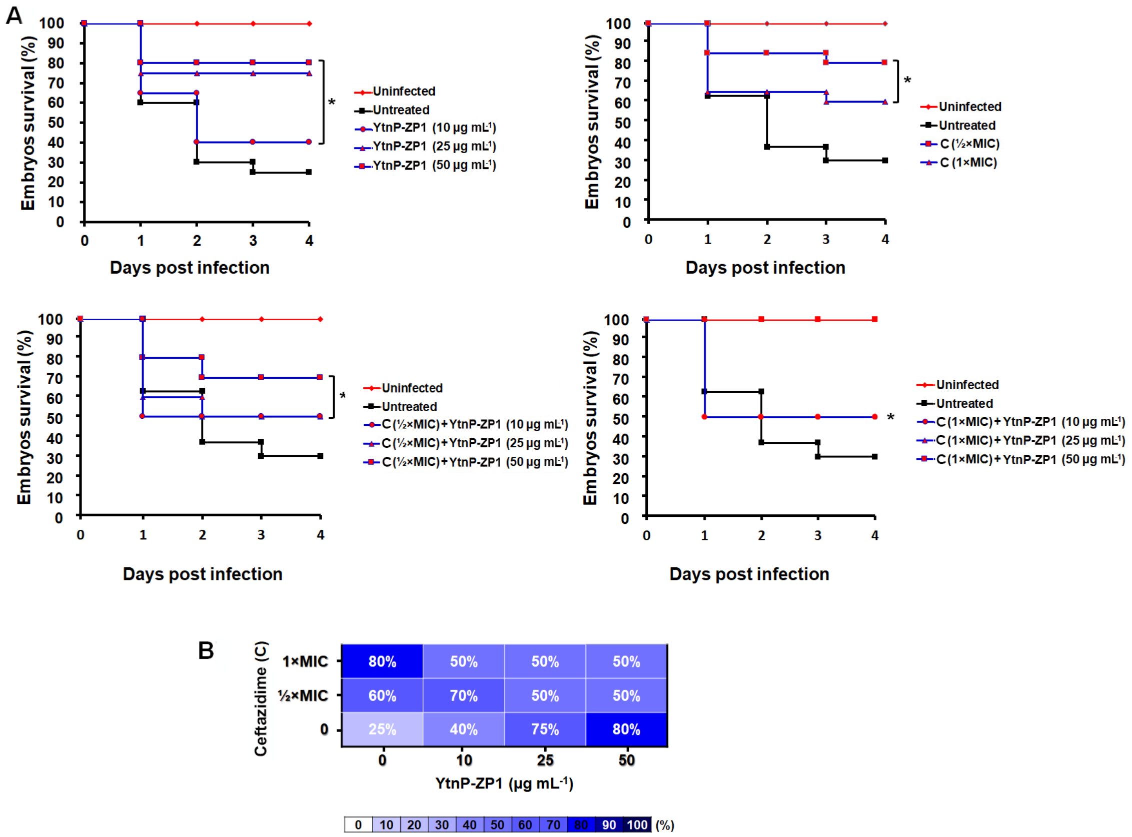

Supplementary Figure 7. Antibacterial efficacy of YtnP-ZP1 (L), ceftazidime (C) and their combinations was assessed in the *P. aeruginosa* PAO1-zebrafish infection model. The Kaplan-Meier survival curves of the untreated and treated infected embryos during four days post infection (A) as well as the cumulative survival rate at the end of the experiment (B) are shown.

2.2 Supplementary Tables

Supplementary Table 1. Primers used in this study

	Sequence
**ZP1LTN F**	GAAGGAGATATACATATGGAAACTTTAACAATCGGACGA
**ZP1LTN R**	GTGATGGTGGTGATGATGGCGCCTTTCTCTTTTTACTGA
**T7 promoter**	TAATACGACTCACTATAGGG
**pETite reverse**	CTCAAGACCCGTTTAGAGGC

Supplementary Table 2. Lethal and teratogenic effects observed in zebrafish (*Danio rerio*) embryos at different hours post fertilization (hpf).

Category	Toxicological parameters	Exposure time (hpf)				
		24	48	72	96	120
**Lethal effect**	Coagulated eggs[a]	•	•	•	•	•
	Lack of the heart beating	•	•	•	•	•
	Non-detachment of the tail	•	•	•	•	•
	Lack of somite formation	•	•	•	•	•
**Teratogenic effect**	Malformation of head	•	•	•	•	•
	Malformation of eyes[b]	•	•	•	•	•
	Malformation of sacculi/otoliths[c]	•	•	•	•	•
	Malformation of chorda	•	•	•	•	•
	Malformation of tail[d]	•	•	•	•	•
	Scoliosis	•	•	•	•	•
	Yolk edema	•	•	•	•	•
	Yolk deformation	•	•	•	•	•
	Growth retardation[e]		•	•	•	•
	Hatching			•	•	•
	Swimmbladder development					•
**Hepatotoxicity**	Yolk absorption			•	•	•
	Liver darkening			•	•	•
**Cardiotoxicity**	Pericardial edema		•	•	•	•
	Heart morphology			•	•	•
	Heart beating rate (beat/min)				•	•
**Melanocytotoxicity**	Skin pigmentation (melanization)[f]		•	•	•	•
	Melanocytes morphology[g]		•	•	•	•

[a]No clear organ's structure is recognized.
[b]Malformation of eyes was recorded for the retardation in eye development and abnormality in shape and size.
[c]Presence of none, one or more than two otoliths per sacculus, as well as reduction and enlargement of otoliths and/or sacculi (otic vesicles).
[d]Tail malformation was recorded when the tail was bent, twisted or shorter than to control embryos as assessed by optical comparation.
[e]Growth retardation was recorded by comparing with the control embryos in a body length (after hatching, at and onwards 72 hpf) using by optical comparison using an inverted microscope (CKX41; Olympus, Tokyo, Japan).
[f] Skin depigmentation of hyperpigemntation was assessed by optical comparation.
[g]Change in stellate morphology of the skin melanocytes was visually recorded

Supplementary Table 3. Hydrolysis of C4 HSL (500 µM) with NaOH solution (20 mM).

	C4 HSL (RT = 5.82 min)		Product (RT = 3.75 min)	
Reaction time	Area (mU*s)	Area (%)	Area (mU*s)	Area (%)
No NaOH	7185.76	100	-	-
10 min	-	-	7667.03	100

Supplementary Table 4. Hydrolysis of C4 HSL (500 µM) with YtnP-ZP1 (10 µg mL^{-1}).

	C4 HSL (RT = 5.8 min)		Product (RT = 3.7 min)		Ratio (%)
Rection time	Area		Area		
	(mU*s)	%	(mU*s)	%	
0 min	7984.17	100.0	-		-
30 min	7396.78	98.1	145.519	1.9	49 : 1
1h	7272.97	95.1	371.214	4.9	19 : 1
2h	6692.72	89.0	829.944	11.0	8 : 1
4h	6191.56	79.0	1643.67	21.0	4 : 1
6h	5961.22	70.3	2516.11	29.7	2.3 : 1
24h	3391.62	31.8	7261.62	68.2	1 : 2
Control (500 µM) C4 HSL in buffer)					
0 min	7901.19	100.0	-		
6h	5864.05	75.3	1923.42	24.7	3 : 1
24h	3321.81	37.9	5442.15	62.1	1 : 1.6

Supplementary Table 5. Hydrolysis of 3-oxo-C12 HSL (500 µM) with NaOH solution (20 mM).

	3-oxo-C12 HSL (RT = 11.2 min)		Product (RT = 10.02 min)	
Reaction time	Area (mU*s)	Area (%)	Area (mU*s)	Area (%)
No NaOH	4005.34	90	442.98	10
10 min	156.04	7.0	2070.28	93.0
20 min	64.60	2.2	2836.93	97.8
30 min	-	-	5410.07	100.00

Supplementary Table 6. Hydrolysis of 3-oxo-C12 HSL (500 µM) with YtnP-ZP1 (10 µg mL^{-1}).

	3-oxo-C12 HSL (RT = 11.2 min)		Product (RT = 10.02 min)		Ratio (%)
Reaction time (min.)	Area		Area		
	(mU*s)	(%)	(mU*s)	(%)	
0	4629.71	93.7	312.45	6.3	14.8 : 1
30	3747.99	77.1	1115.64	22.9	3.4 : 1
60	2461.63	47.3	2738.12	52.7	1 : 1.1
90	1652.45	29.3	3994.68	70.7	1 : 2.4
120	919.37	16.8	4566.82	83.2	1 : 5
150	462.51	8.3	5089.82	91.7	1 : 11
180	378.93	6.6	5362.48	93.4	1 : 14.1
210	280.37	4.8	5557.79	95.2	1 : 19.8
240	197.27	3.2	5893.57	96.8	1 : 29.9
300	-	0	6583.82	100	-
Control (500 µM) 3-oxo-C12 HSL in buffer					
0 min	4770.84	97.1	141.04	2.9	33.5 : 1
6h	2771.42	70.4	1164.27	29.6	2.4 : 1
24h	1986.64	48.4	2114.65	51.6	1 : 1.1

Supplementary Table 7. Inhibition of biofilm formation (%) in environmental isolates with 50 µg mL^{-1} of YtnP-ZP1 (L). Biofilms of environmental isolates formed in the absence of YtnP-ZP1 (C). The results represent the average of three independent experiments performed for each strain ± SD. Isolates with > 40% inhibition of biofilm formation by YtnP-ZP1 are bolded.

Isolates	A_{570nm}		% of inhibition
	C	L	
**KS+3**	**3.67±0.06**	**0.56±0.23**	**84.81±6.13**
**KM+3**	**0.80±0.15**	**0.38±0.07**	**52.09±8.47**
KS-5	0.87±0.08	0.70±0.11	25.16± 5.04
KS+5	0.58±0.04	0.78±0.24	no inhibition
KM-5	2.54±0.18	2.97±0.13	no inhibition
KS-6	0.91±0.10	0.80±0.06	13.18±4.84
**KS-7**	**1.14±0.06**	**0.35±0.04**	**69.40±3.31**
**KS+8**	**0.44±0.05**	**0.011±0.01**	**74.27±1.83**
KM-9	2.12±0.15	1.91±0.25	15.23±3.25
**KS+13**	**0.64±0.07**	**0.37±0.04**	**42.72±6.30**
KS-17	1.16±0.06	0.91±0.08	21.19±6.49
KS-18	0.69±0.09	0.66±0.09	no inhibition
KS-20	1.08±0.04	0.75±0.05	30.57±4.95
KS-23	1.17±0.05	0.87±0.07	26.08±5.67
KS-26	1.04±0.06	1.14±0.07	no inhibition
KS-27	0.87±0.10	0.82±0.12	10.62±5.81
**KS-112**	**1.54±0.12**	**0.58±0.08**	**62.01±5.35**
**KM-151**	**1.76±0.81**	**0.19±0.03**	**89.81±1.00**

Supplementary Table 8. Phenotypic characterization of environmental isolates with the highest inhibition of biofilm formation by YtnP -ZP1.

Isolate	*Pseudomonas* sp. KS+3	*Brucella* sp. KM+3	*Sphingobacterium* sp. KS-7	*Agrobacterium* sp. KS+8	*Brucella* sp. KS+13	*Pseudomonas* sp. KS-112	*Serratia* sp. KM-151
Catalase	+	+	+	+	+	+	+
Cellulase	+	-	+	-	-	+	+
Lipase	+	-	-	-	+	-	+
DNase	-	-	-	-	-	-	+
Hemolytic activity	α	-	-	α	-	-	-
EPS production	-	-	+	-	-	+	-
Urea hydrolysis	+	+	+	+	+	+	-
Starch hydrolysis	+	-	+	-	-	+	+
Citrate utilization	+	+	+	+	+	+	+
Glucose	+	+	+	+	+	+	+
Fructose	+	+	+	+	+	+	+
Lactose	+	+	+	+	+	+	-
Sucrose	+	+	+	+	+	+	+

Supplementary Table 9. Minimum inhibitory concentration (MIC) and minimum bactericidal concentration (MBC) of clinically relevant antibiotics in the absence or presence of YtnP-ZP1 (50 µg mL^{-1}) after 20 h treatment of *P. aeruginosa* PAO1.

**Antibiotic**	**Without YtnP-ZP1 MIC/MBC (**µg mL^{-1})	**WithYtnP-ZP1 MIC/MBC (**µg mL^{-1})
Tobramycin	4/4	4/4
Gentamicin	2/2	2/2
Ceftazidime	1//32	1/32

Supplementary references

Mihajlovic, L.E., Savic, A., Poljarevic, J., Vuckovic, I., Mojic, M., Bulatovic, M., Maksimovic-Ivanic, D., Mijatovic, S., Kaluderovic, G.N., Stosic-Grujicic, S., *et al.* (2012). Novel methylene modified cyclohexyl ethylenediamine-N,N'-diacetate ligands and their platinum(IV) complexes. Influence on biological activity. *J Inorg Biochem* 109, 40-48.

Plou, F.J., Ferrer, M., Nuero, Calvo, M.V., Alcalde, M., Reyes, F. & Ballesteros, A. (1998) Analysis of Tween 80 as an esterase/lipase substrate for lipolytic activity assay. *Bio Tech* 12, 183-186.

Kasana, R.C., Salwan, R., Dhar, H., Dutt, S. and Gulati, A. (2008) A rapid and easy method for the detection of microbial cellulases on agar plates using Gram's iodine, *Curr Microbiol* 57, 503-507.

Bailey & Scott's Diagnostic Microbiology, 13th Edn, Edited by Patricia M. Tille. St. Louis: Mosby, Inc, 2014. ISBN: 978-0-323-08330-0

Lal, A. and Cheeptham, N. (2012) Starch agar protocol. American Society for Microbiology © 2016.
